# PhyEffector, the First Algorithm That Identifies Classical and Non-Classical Effectors in Phytoplasmas

**DOI:** 10.3390/biomimetics8070550

**Published:** 2023-11-17

**Authors:** Karla Gisel Carreón-Anguiano, Sara Elena Vila-Luna, Luis Sáenz-Carbonell, Blondy Canto-Canche

**Affiliations:** Unidad de Biotecnología, Centro de Investigación Científica de Yucatán, A.C., Calle 43 No. 130 x 32 y 34, Colonia Chuburná de Hidalgo, Mérida C.P. 97205, Yucatán, Mexicosaravilalu@gmail.com (S.E.V.-L.); vyca@cicy.mx (L.S.-C.)

**Keywords:** phytoplasmas, crop pathogens, PhyEffector algorithm, classical and non-classical effectors

## Abstract

Phytoplasmas are the causal agents of more than 100 plant diseases in economically important crops. Eleven genomes have been fully sequenced and have allowed us to gain a better understanding of the biology and evolution of phytoplasmas. Effectors are key players in pathogenicity and virulence, and their identification and description are becoming an essential practice in the description of phytoplasma genomes. This is of particular importance because effectors are possible candidates for the development of new strategies for the control of plant diseases. To date, the prediction of effectors in phytoplasmas has been a great challenge; the reliable comparison of effectoromes has been hindered because research teams have used the combination of different programs in their predictions. This is not trivial since significant differences in the results can arise, depending on the predictive pipeline used. Here, we tested different predictive pipelines to create the PhyEffector algorithm; the average value of the F1 score for PhyEffector was 0.9761 when applied to different databases or genomes, demonstrating its robustness as a predictive tool. PhyEffector can recover both classical and non-classical phytoplasma effectors, making it an invaluable tool to accelerate effectoromics in phytoplasmas.

## 1. Introduction

Phytoplasmas are pleomorphic, mycoplasma-like bacterial pathogenic microorganisms. These organisms are between 0.2 to 0.8 µm in diameter and lack cell walls; they reside in plant phloem and are transmitted by phloem-feeding insect vectors [1,2,3]. Phytoplasmas cause severe damage to the agriculture and horticulture industries worldwide, with extensive yield losses in thousands of economically important crops such as vegetables, spices, medicinal plants, ornamentals, palms, fruit trees, among others [4]. Infected plants display symptoms such as phyllody (transformation of floral organs into tissues similar to leaves), virescence (green coloration in floral organs), yellowing (chlorosis), stunting, Little Leaf disease, and witches’ broom (proliferation of shoots) [5,6]. Phytoplasmas cause these abnormal plant morphologies through the secretion of virulent proteins known as effectors, which interfere with the hormone signaling of their hosts [7,8].

Effectors are defined as small molecules that selectively bind to a protein and regulates its biological activity [9]. These molecules are fundamental in the parasite–host interaction, where the parasite is metabolically dependent on its host; a struggle of forces is established between the attack of the parasite and the defense of the host. Effectors play key roles in the successful infection of the plant host by the pathogen [10].

To date, eleven phytoplasma genomes have been sequenced (Cho et al. (2020) [11]; KEGG, https://www.genome.jp/kegg/genome/; accessed on 10 July 2023), ranging from ~570 Mb to ~950 Mb, and comprising ~470 to 870 proteins [12]. The number of predicted effectors reported per genome ranges from 10 in the genome of ‘*Ca*. Phytoplasma asteris’ De Villa [11], 65 effectors in the genome of the AY-WB strain OY-M [13], and 98 in ‘*Ca*. Phytoplasma aurantifolia’ [14].

Phytoplasma effectors are secreted proteins, usually with no transmembrane domains. However, although this description of the effectors is relatively simple, their identification has followed different routes. For signal peptide identification, different versions of SignalP have been used, a program that has been trained to predict the presence of signal peptides (SP) in gram-negative or gram-positive bacterial proteins. Bai et al. (2009) [15] evaluated SignalP v3.0 on 369 mollicute protein sequences that included 46 experimentally verified secreted proteins; the program was able to identify 43 of these 46 secreted proteins. After using SignalP v3.0, and eliminating the SP (20–50 amino acids) sequence, the proteins were analyzed using the TMHMM v2.0 program to identify and eliminate proteins with transmembrane domains (TMDs). Using this approach, 56 effector candidates were found in the genome of the aster yellows phytoplasma strain witches’ broom.

Anabestani et al. (2017) [16] used a similar approach, but compared SignalP v3.0 and SignalP v4.0, selecting the latter as the predictor for their secreted proteins; they predicted 28 effectors in ‘*Ca*. Phytoplasma aurantifolia’, nine of them with nuclear localization. Wang et al. (2018) [17] used the same pipeline, combining SignalP v4.0 and TMHMM v2.0 after eliminating the SP and found 28 effectors in the Jujube Witches’ Broom (JWB) Phytoplasma genome. Recently, Tan et al. (2021) [14] compared the use of SignalP v4.1 vs. SignalP v5.0. With the latter they found 28 extracellular proteins, while SignalP v4.1 excluded one of these proteins, but retrieved 70 additional secreted proteins. However, they found by manual analysis that many of these proteins were false positives, warning users about the accuracy of the predictions depending on the version of SignalP used. Previously, Cho et al. (2020) [11] used SignalP v5.0 (in Gram-positive bacterial mode), then filtered with TMHMM v2.0 to retain the secreted proteins with no TMDs; they analyzed 11 phytoplasma genomes and found differences in the results previously reported by Music et al. (2019) [18] using SignalP v3.0. For example, these authors predicted eight effectors for AYWB strain, and 15 effectors for OY-M strain, while Cho et al. (2020) [11] predicted 33 and 37 effectors, respectively.

Most of the previous pipelines focused on predicting effectors as secreted proteins with no TMDs. However, a few known phytoplasma effectors are transmembrane proteins, such as Amp [19] and Imp [20]. Very recently, Debonneville et al. (2022) [12] combined the use of SignalP v5.0 with Phobius to predict the presence of signal peptide and TMDs in effectors from the ‘Flavescence Dorée’ Phytoplasma. This combination identified 17 effector candidates; Phobius identified three secreted proteins with a TMD and a SP. In addition, Phobius was able to retrieve two putative secreted proteins that are unique to the genome of this phytoplasma, which suggests that Phobius must be considered in pipelines for the identification of effectors in phytoplasmas.

Interestingly, Gao et al. (2023) [21] identified 95 non-classically secreted proteins (ncSecPs) in the ‘*Ca*. Phytoplasma ziziphi’ genome for the first time and confirmed that 25 of them are true secreted proteins. Non-classically secreted proteins lack SPs or translocation signals, but are still exported to the extracellular space through a Sec-independent secretion pathway. These proteins are not identified by the SignalP program but by SecretomeP. These novel results show that classic pipelines for the identification of phytoplasma effectors must be revised and innovated.

Here, we performed a comparison of different pipelines comprising either SignalP v3.0, SignalP v4.1 or SignalP v5.0, with TMHMM v2.0 (on SP-lacking proteins), followed by sequential analysis with Phobius, SecretomeP and sequence-homology-based Blastp. The false positives retrieved by any of the steps were eliminated. The performance of the pipelines was evaluated on positive and testing datasets (comprising phytoplasma effectors) and negative datasets (phytoplasma non-effectors proteins), resulting in F1 scores from 0.56 to 1.0. The best algorithm was named PhyEffector and it was further evaluated on phytoplasma genomes (i.e., deduced proteomes), resulting in an average F1 score of 0.9761. These results showed that PhyEffector is a robust predictor of phytoplasma effectors, with very low false negatives (generally < 10). PhyEffector is able to retrieve both classical effectors (containing a SP and without TMD) as well as non-classical (atypical) effectors from phytoplasmas (effectors that contain a TMD or lack SP and are secreted by a Sec-independent secretion pathway), which makes PhyEffector a powerful computational tool to speed up phytoplasma effectoromics.

## 2. Materials and Methods

### 2.1. Creation of Databases

Positive dataset: The positive dataset was constructed with 10 of 21 validated phytoplasma effectors reported in the literature (Table 1): 5 of them canonical effectors (secreted and identified by any version of the SignalP program) and 5 non-canonical ones (with a TDM, or secreted by a non-classical pathway). The list was completed by searching in the UNIPROT database with the keywords: SAP01, SAP02, SAP03, until SAP80; TENGU, phyllody, phyll, antigenic membrane protein (Amp), Immunodominant membrane protein A (idpA), Immunodominant membrane protein (Imp), and Variable membrane protein A (VmpA). The complete retrieved list contained 738 protein sequences. To prevent over-representation due to large effector families, 4 members were chosen per family, except for SAP11, for which sequence identity among them was lower than 45%. Similarly, to avoid over-fitting due to highly conserved effector sequences, members with ~50% sequence identity were selected, except for largely conserved families such as TENGU, idpA and PME. The final list of positive datasets of phytoplasma effectors comprised 64 sequences (Table S1).

A second database was constructed for testing PhyEffector, comprising the other 11 true effectors (6 canonicals and 5 non-canonicals), plus 215 of the other potential effectors retrieved from UNIPROT. These 226 potential effectors share ~50% identity with protein sequences in the positive database.

Negative dataset: Core phytoplasma proteomes were identified after manual revision of phytoplasma genomes available at KEGG (https://www.genome.jp/kegg/genome/; accessed on 15 July 2023). Conserved KEGG pathways were identified, such as: glycolysis/gluconeogenesis, citrate cycle (TCA cycle), pentose phosphate pathway, fructose and mannose metabolism, purine metabolism, pyrimidine metabolism, glycine, serine and threonine metabolism, valine, leucine and isoleucine degradation, etc. Among these KEGG pathways, 64 core protein sequences with 40 different functional annotations were used to construct the negative dataset (Table S2).

### 2.2. In Silico Characterization of Effectors from Phytoplasmas

To gain knowledge about the features of currently known phytoplasma effectors, the positive dataset was analyzed with SignalP v4.1, TMHMM v2.0 on mature proteins (without signal peptide), Phobius and SecretomeP v2.0.

### 2.3. Characterization of Different Pipelines to Predict Effectors in Phytoplasmas: Construction of PhyEffector Algorithm

Different versions of SignalP: v3.0 [22], 4.1 [23] and 5.0 [24] were tested independently in Gram-positive and Gram-negative modes to identify secreted proteins, and after removal of the SP, the proteins were filtered with TMHMM v2.0 [25], resulting in each case in the set #1.

**Figure 1:**
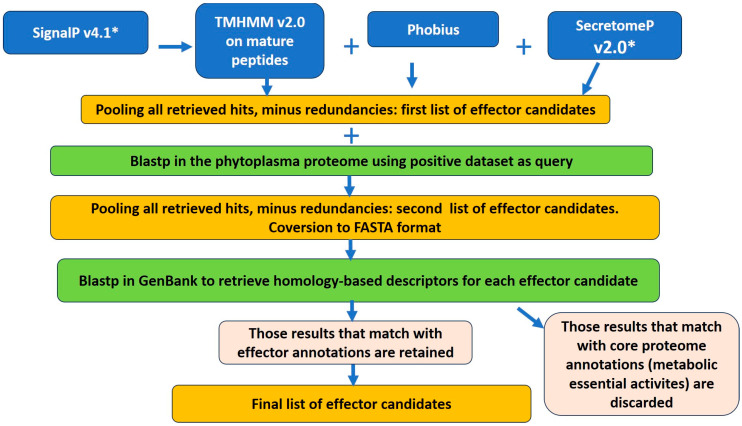
PhyEffector workflow for the prediction of effectors in phytoplasma proteomes. *, programs are run in Gram-positive mode. Mature proteins are signal peptide-lacking proteins. Blue squares, pre-installed prediction tools; green squares, Blastp analyses; pink squares, custom tools for filtering results based on descriptors; yellow squares, lists of effector candidates. All databases were constructed here, except the database of annotations of metabolic essential activities, which was based on the open access report from Cho et al. (2020) [11].

Prediction by Phobius server (set #2) [26], SecretomeP (set # 3) [27] and Blastp analysis using the dataset of phytoplasma effectors as query (set # 4) were pooled, and then redundancies were eliminated in the results of each pipeline, becoming the “total potential effectorome” for each pipeline. These proteins were annotated performing a Blastp against the GenBank non-redundant protein database (https://www.ncbi.nlm.nih.gov/; accessed on 10 August 2023); proteins annotated as hypothetical proteins, unknown function, predicted proteins, no hits, as well as pathogenic/virulence related functions and those with annotations related to phytoplasma effectors, were selected and comprise the final list of effectors, while proteins with metabolic essential functions were discarded (Figure 1). Table S3 shows the list of functional descriptors (annotations) of known effectors. The list of annotations of essential metabolic activities was obtained from Cho et al. (2020) [11].

**Table 1:** List of true phytoplasma effectors used in the present work.

Effector	Accession at GenBank/UNIPROT	Homolog	Phytoplasma	Phenotype or Function	Observations	Dataset	Reference
	Canonical, typical or classical						
TENGU	BAH29766.1/A0A4P6MDK8	------	‘*Ca*. Phytoplasma Asteris’, strain Onion yellows phytoplasma OY-M. Group 16SrI.	Dwarfism, witches’ broom symptoms and plant sterility. Pleiotropic effects on auxin and jasmonic acid	First reported witches’ broom-inducing effector. Small protein (70-amino acid preprotein, of which 38 C-terminal amino acids are released into plant host)	Positive set	[28,29]
SAP05	8PFC_A WP_011412316.1	------	Aster Yellows phytoplasma strain Witches’ Broom (AY-WB)	Induces witches’ broom symptoms, Proliferation of vegetative tissue and shoots.	Binds plant SPL and GATA transcription factors and mediates their degradation in a ubiquitin-independent manner	Positive set	[30]
SAP11	GI:85057650	------	Aster Yellows phytoplasma strain Witches’ Broom (AY-WB). Crinkled leaves and siliques	CIN-TCP binding and destabilization, and impaired synthesis of jasmonic acid, and increase in leafhopper oviposition activity.	Modular organization; at least three domains are required for efficient CIN-TCP destabilization in plants	Positive set	[31]
SAP54	WP_252861407.1	------	Aster Yellows phytoplasma strain Witches’ Broom (AY-WB). Virescence	Degrading MADS-box Proteins; induces phyllody and sterile plants	-------	Positive set	[32]
PHYL1	LC388988.1, LC3889891, LC3889911, LC388990.1, LC388981.1, LC388982.1, LC388983.1, LC388992. 1, LC492887.1, LC388972.1, LC388985.1, LC388987.1	SAP-54	“*Ca*. Phytoplasma” species	Witches’ broom symptoms	Phyllogens (four groups: phyl-A, -B, -C, and -D)	Positive set	[33]
SWP1	WP_024563292.1	SAP11-like	Wheat blue dwarf phytoplasma	witches’ broom symptoms	------	Testing set	[17]
SWP11	No GenBank accession. Arbitrary authors’ code WBD_0004	------	Wheat blue dwarf phytoplasma	Cell death and defence responses, including H_2_O_2_ accumulation and callose deposition.	Up-regulation of HIN1, PR1, PR2 and PR3	Testing set	[34]
SWP12	No GenBank accession. Arbitrary authors´ code WBD_0238	------	Wheat blue dwarf phytoplasma	suppress SWP11-, BAX-, and/or INF1-induced cell death	------	Testing set	[34]
SWP21	No GenBank accession. Arbitrary authors´ code WBD_0274	TENGU-like	Wheat blue dwarf phytoplasma	suppress SWP11-, BAX-, and/or INF1-induced cell death	SWP21 has a distinct role in virulence compared with TENGU	Testing set	[29,34]
Zaofeng3	AYJ01078.1	SAP54-like	‘*Ca*. Phytoplasma ziziphi’ (JWB phytoplasma) (16SrV-B)	Overexpression showed phytoplasma-like symptoms	87% identity with SAP54PnWB	Testing set	[35]
Zaofeng6	AYJ01297.1	SAP11-like	JWB phytoplasma	Overexpression resulted in shoot proliferation; triggered hypersensitive response and induced the expression of defense-related genes	48% identity with SAP11AYWB.	Testing set	[35]
	Non-canonical, atypical or non-classical						
IdpA	ADD52250.1	------	Poinsettia branch-inducing phytoplasma	Crucial role in plant and insect vector transmission	Immunodominant membrane protein A; transmembrane domain present	Positive set	[36]
Imp	CBJ17020.1	------	‘*Ca*. Phytoplasma mali’	Binds to plant actin; probably involved in phytoplasma motility in host plants	Immunodominant membrane protein; transmembrane domain present	Positive set	[20]
VmpA	ULR56812.1	------	Flavescence dorée phytoplasma	Binds the midgut of the insect vector and promotes adhesion to its epithelial cells.	Variable membrane protein A; transmembrane domain present	Testing set	[37]
Amp	WP071345415.1	------	Rice orange leaf Phytoplasma	Suppresses host defenses. Interacts with actin of its vector; probably involved in vector specificity	Antigenic membrane protein; transmembrane domain present	Positive set	[38]
ncSecP3	WP_161554967.1	------	‘*Ca*. P. ziziphi’	Suppresses hypersensitive cell death response (HR) in *Nicotiana bentamiana*, triggered by the pro-apoptotic mouse protein Bax and the *Phytophthora infestans* elicitin INF1	Non-classically secreted proteins (ncSecPs); non-secreted by Sec-pathway	Positive set	[21]
ncSecP9	WP_121463838.1	------	‘*Ca*. P. ziziphi’	Suppresses HR in *Nicotiana bentamiana*, triggered by Bax and INF1	ncSecPs	Positive set	[21]
ncSecP12	WP_161554974.1	------	‘*Ca*. P. ziziphi’	Suppresses HR in *Nicotiana bentamiana*, triggered by Bax and INF1	ncSecPs	Testing set	[21]
ncSecP14	WP_121463915.1	------	‘*Ca*. P. ziziphi’	Suppresses HR in *Nicotiana bentamiana*, triggered by Bax and INF1	ncSecPs	Testing set	[21]
ncSecP16	WP_161554978.1	------	‘*Ca*. P. ziziphi’	Suppresses HR in *Nicotiana bentamiana*, triggered by Bax and INF1	ncSecPs	Testing set	[21]
ncSecP22	WP_121463976.1	------	‘*Ca*. P. ziziphi’	Suppresses HR in *Nicotiana bentamiana*, triggered by Bax and INF1	ncSecPs	Testing set	[21]

These pipelines were run on the positive dataset (phytoplasma effectors) and the negative dataset (non-effector phytoplasma proteins). True positives (TP), false positives (FP), true negatives (TN) and false negatives (FN) were determined to calculate sensitivity, specificity, precision and accuracy parameters. F1 scores were calculated to measure and compare performances of the pipelines.

### 2.4. Validation of PhyEffector Algorithm

PhyEffector was used to carry out the identification of effector candidates on a testing database comprising 11 true effectors (different from those 10 true effectors in the positive dataset) and the 385 protein sequences retrieved from UNIPROT, which shared ~50% identity with protein sequences in the positive dataset.

PhyEffector was validated through the identification of effectors in the genomes (i.e., deduced proteomes) of ‘*Ca*. P. asteris’ AYWB (16SrI-A), ‘*Ca*. P. asteris’ OY-M (16SrI-B), ‘*Ca*. P. aurantifolia’ (16SrII), ‘*Ca*. P. ziziphi’ Jwb-nky (16SrV-B), ‘*Ca*. P. vitis’ of Flavescence dorée phytoplasma’ (16SrV-C), ´*Ca*. P. luffae’ (16SrVIII), ‘*Ca*. P. mali’ (16SrX), ‘*Ca*. P. australiense’ PAa (16SrXII) and ‘*Ca*. P. solani’ SA-1 (16SrXII); prediction by PhyEffector on each phytoplasma genome was compared with the results from the respective report in the literature. Discrepancies between the prediction from PhyEffector and the scientific literature were solved by determining the number of FP and FN for each prediction by following the criteria described above, i.e., identifying those FN using the list of effector annotations, and those FP with the list of annotations of essential metabolic activities.

## 3. Results

### 3.1. Construction of Positive Dataset

The positive dataset was constructed with 21 validated phytoplasma effectors reported in the literature (Table 1), 11 of them canonical effectors (secreted, with SP and no TMD), and 10 non-canonical ones (with a TMD, or secreted by a non-classical pathway). The first attempt to complete the list of phytoplasma effectors was done through searches in GenBank using certain keywords (for example effector names), but results were highly redundant. For example, “phytoplasma effector sap11” retrieved 71 results, but 18 sequences for SAP11 effector protein from ‘Lime witches’-broom phytoplasma (IDs from QAB44970.1 to QAB44987.1) were 100% identical. High redundancy was observed as well in other results while “phytoplasma effector TENGU” retrieved no results from the GenBank (10 March 2023). Phytoplasma effectors were then searched for in the UNIPROT database using the keywords SAP01, SAP02, SAP03, until SAP80; other keywords used were TENGU, phyllody, phyll, antigenic membrane protein (Amp), Immunodominant membrane protein A (idpA), Immunodominant membrane protein (Imp) and Variable membrane protein A (VmpA). The first list of phytoplasma effectors comprised 738 amino acid sequences. The list was carefully revised to eliminate potential false positives; 229 effector candidates annotated as “Candidatus effector” or “phytoplasma effector” were eliminated because they do not share identity with any known phytoplasma effectors and none of them have been experimentally validated. The preferred proteins were phytoplasma effectors with ~50% identity shared among each other to avoid over-representation or over-fitting of effector families; only 15% of the sequences share > 80% identity. The final list comprised 64 phytoplasma effectors (Table S1).

### 3.2. Characterization of Phytoplasma Effectors

The positive dataset was analyzed to classify the phytoplasma effectors in classical (secreted, with SP, with no TMD), and non-classical candidates (those that do not meet any of the characteristics of classical phytoplasma effectors) (Table 2). Most of the known phytoplasma effectors are predicted to be secreted through the canonical type II secretion system. From the other ~10%, half are predicted to be secreted by the non-classical pathway and the other half are predicted as non-secreted. Understandably, it was found that almost 92% were predicted to have no TMD, while ~8% have one TMD, but the occurrence of TMD indicates that a “no TMD” criterion leads to the underestimation of the phytoplasma effectoromes.

### 3.3. Comparison of Multiple Pipelines to Identify Phytoplasma Effectors

The most common pipelines for the identification of phytoplasma effectors use SignalP v4.1 or SignalP v5.0 and TMHMM v2.0 on SP-lacking mature proteins [11,16,18,21,36]. Only a few reports have modified these pipelines to include other programs such as Phobius [12] and SecretomeP v2.0 [21]. Most authors have used SignalP programs in Gram-positive mode [11,16,18,21,36]. Recently, Gao et al. (2023) [21] argued that phytoplasmas have a distinct membrane composition, and they are neither in the Gram-positive group nor the Gram-negative group; these authors used both SignalP program modes to identify effectors in ‘*Ca*. Phytoplasma ziziphi’. Based on these reports, different pipelines were tested in our analyses using SignalP v4.1 or SignalP v5.0 in either Gram-positive or Gram-negative modes.

Taking into consideration that ~8% of phytoplasma effectors were found to have TMDs, and ~10% were elusive to the SignalP programs (Table 2), the pipelines constructed here included additional programs, Phobius and/or SecretomeP. The results of these programs were pooled and redundancies were eliminated. Table 3 shows the performance of pipelines with programs used in Gram-positive mode, and Table 4 for Gram-negative mode.

The pipelines were constructed as follows:

1. Signalp4.1 + phobius + secretomeP2.0 + TMHMM2.0
2. Signalp4.1 + phobius + secretomeP2.0 + TMHMM2.0 + BLASTP+ elimination of false positive
3. Signalp4.1 + phobius + TMHMM2.0
4. Signalp4.1 + phobius + TMHMM2.0 + BLASTP+ elimination of false positive
5. Signalp5.0 + phobius + secretomeP2.0 + TMHMM2.0
6. Signalp5.0 + phobius + secretomeP2.0 + TMHMM2.0 + BLASTP+ elimination of false positives
7. Signalp5.0 + phobius + TMHMM2.0
8. Signalp5.0 + phobius + TMHMM2.0 + BLASTP+ elimination of false positives

For both classes of pipelines, either with SignalP v.4.1 or SignalP v5.0, the Gram-negative mode achieved F1 scores lower than their counterpart in the Gram-positive mode. These results indicate that the Gram-positive mode is more suitable for the prediction of phytoplasma effectors. The pipelines with SignalP + TMHMM + Phobius retrieved consistently fewer false positives (pipelines 3 and 7) compared to pipelines that included these programs plus SecretomeP v2.0 (pipelines 1 and 5), but also excluded more effectors in comparison with the pipelines that include SecretomeP (see pipelines 3 and 7 and compare with pipelines 1 and 5). These results support pipelines 1 and 5 to continue the analysis. Pipelines 1 and 5 differ in the version of SignalP in use. The pipeline that includes SignalP v4.1 retrieved 16 more false positives (7) than retrieved by SignalP v5.0 (3), but the pipeline with SignalP v5.0 excluded 9 effectors (false negatives) while the pipeline with SignalP v4.1 excluded 3; in other words, pipeline 1 is able to retrieve 6 effectors that pipeline 5 could not (Table 3). The balance of false positives and false negatives lead to an F1 score of 0.91 for pipeline 1, and an F1 score of 0.90 for pipeline 5.

To improve the performance of pipeline 1, three databases were independently linked for the effector identification: (A) the positive dataset (Table S1), which was compiled with the results of the Blastp analysis and contains the potential homologs of known effectors; these results were included as effector candidates. (B) a list of annotations/functional descriptors of known effectors (Table S3); this step provides additional supports for effector candidates (reinforces true positives). When some predictors fail to identify “true positive” effectors, the outcome is “false negatives” for that predictor.

(C) a list of 40 annotations/descriptors of essential metabolic activities (the list of these annotations was obtained from KEGG) (Table S2). This step allows for the exclusion of false positives.

### 3.4. PhyEffector Pipeline

Based on the performance of the pipelines analyzed above to identify phytoplasma effectors, the PhyEffector pipeline was constructed with SignalP v4.1, TMHMM v2.0, Phobius and SecretomeP v2.0. Their results were pooled, redundancies were eliminated, and the candidates were subsequently added to the results from Blastp using the positive dataset as query. This second list of effector candidates was converted to FASTA format and submitted to Blastp against the non-redundant protein database at GenBank to obtain homology-based descriptions of all the retrieved hits. Their descriptions were then compared with the list of functional descriptions of known phytoplasma effectors (Table S3). Those hits that did not have any effector-related annotations were then further compared with the list of functional annotations of metabolic essential activities (proteins that correspond to phytoplasmas´ core proteome); those which matched with descriptions in this set were discarded. Figure 1 shows the complete PhyEffector workflow.

### 3.5. PhyEffector Performance: Prediction of Effectors on a Testing Dataset and on Phytoplasma Genomes and Comparison with Literature

A second validation of the different pipelines was conducted on a testing dataset composed of a different set of 11 validated effectors, along with 192 proteins annotated in UNIPROT as “*Candidatus* effector” or “phytoplasma effector” and 23 homologs of the proteins comprising the positive dataset to give 226 proteins in total (Table S4). These protein sequences share ~50% identity with each other in the testing dataset (except for effector families which are largely conserved) and ~50% identity with protein sequences from the positive dataset. Those annotated as “effectors” are largely divergent from each other and share no identity with the positive dataset. A second negative dataset, comprising 226 core proteins (Table S5), different from the first negative dataset, was constructed as well. Validation of PhyEffector on these sets calculates a realistic F1 score since positive and testing datasets comprise different proteins with low identity among them. Table 5 shows the performance of PhyEffector on the testing dataset, as well as the comparison with the other pipelines, to re-analyze whether pipeline # 2 (denominated PhyEffector) is the best predictor for phytoplasma effectors. The F1 score of PhyEffector was 0.90 and it was higher than in all the other pipelines, reinforcing that this pipeline is the best option for further analyses.

In a third analysis for the validation of PhyEffector, the algorithm was used to identify effectors in phytoplasma genomes (i.e., deduced proteomes), and the results were compared with corresponding scientific reports. In order to solve discrepancies between the prediction of PhyEffector and the literature, and to determine which prediction is stronger, false negatives (FN) were identified comparing the annotations of the predicted effectors with the descriptions of known effectors; those that coincide with “true effector” annotations were considered “true”, and those that were not recognized (i.e., lacking effector-associated annotations) were considered “FN”. False positives (FP) were identified with a similar strategy, but instead, the effector results were compared with functional annotations of essential (core) proteins; those that coincided with essential proteins were considered “false positives”.

The number of predicted effectors by PhyEffector was highly variable, from 41 in the Flavescence dorée phytoplasma, to 97 effector candidates in ‘*Ca*. Phytoplasma asteris’ DY2014. In all cases, PhyEffector predicted a higher number of effector candidates than the scientific reports (Table 6). Since the prediction by PhyEffector included the FP elimination step, the number of FP was zero in all predictions, while FN were less than 10 in general, except in ‘*Ca*. Phytoplasma asteris’ (AY-WB), where PhyEffector had 17 FN. However, the predictions from the corresponding literature had higher numbers of FN and higher numbers of FP. Based on the results obtained from the various phytoplasma genomes, the F1 score for PhyEffector ranked from 0.8957 to 1.0, with an average value of 0.9783. These results indicate that the PhyEffector is a robust algorithm to identify effector candidates in phytoplasmas.

For this evaluation, the proteins that were not retrieved by the pipelines from the “Testing set” were considered as “false negatives” for F1 score determination. However, these proteins are actually “ambiguous” since they may be false positives and the PhyEffector´ F1 score may be higher for each pipeline in Table 5.

## 4. Discussion

Effectors are essential for virulence and are therefore susceptible targets for the control of phytoplasma-associated diseases [39,40]. It is necessary to have a robust computational tool that specifically identifies these effector candidates to accelerate phytoplasma effectoromics.

A positive database (phytoplasma effectors) was constructed by searching with keywords such as “Phytoplasma effectors”, “SAP effector”, etc. The first attempt was the search in the GenBank database, but it resulted in highly redundant data. Better results were obtained in the UNIPROT database, but manual revision was also necessary. Finally, the positive dataset comprised 64 non-redundant proteins. Difficulties involved in the prediction of effectors include the small number of true (validated) currently known effectors, and the number of potential false positives in the public databases. We found that many proteins annotated in UNIPROT as “phytoplasma effectors” and “*Candidatus* effectors” correspond to “hypothetical proteins” in GenBank. Although many hypothetical proteins are expected to be effectors, it cannot be ruled out that false positives are among them. Here, care was taken and we did not include proteins annotated as “effectors” (phytoplasma effectors or *Candidatus* effectors) according to UNIPROT, which have no homology to known validated effectors and have no experimental validation either, in the positive dataset. However, they were included in another dataset to test the algorithm. Those from the testing dataset that were not retrieved by PhyEffector were counted as false negatives, although they are ambiguous because it is not possible to classify them as effectors or non-effectors at this time.

We reasoned that, if the positive dataset comprises phytoplasma effectors, which are dispensable proteins, i.e., most of them are not ubiquitous in all phytoplasmas, and have a patchy phylogenetic distribution [41,42], the negative dataset must comprise indispensable proteins from phytoplasmas. Indispensable proteins are widely distributed in phytoplasmas, and are related to essential metabolic activities (part of the core proteome). Music et al. (2019) [18] compared the proteomes of phytoplasmas from 16SrI, 16SrXII and 16SrXVI and found 259 orthologous gene clusters shared among them. Recently, Cho et al. (2020) [11] compared 11 genomes of 16SrI-phytoplasmas and found 303 single-copy genes shared among all of them. This list is representative of the core proteome in phytoplasmas and the size is similar to the extended negative dataset constructed in the present study.

Usually, phytoplasma effectors are described as secreted proteins, but recent reports have uncovered the existence of phytoplasma effectors having transmembrane domains (TMDs) or being secreted by a non-classical pathway [21]. Analyzing the positive dataset (64 phytoplasma effector proteins) showed that these proteins are more common than previously believed: ~8% of the phytoplasma effectors have TMDs and ~5% are non-secreted. Based on these findings, different pipelines comprising different predicted programs were constructed and evaluated.

Gram-positive bacteria are surrounded by a thick peptidoglycan cell wall, while Gram-negative bacteria have a much thinner peptidoglycan cell wall with an outer membrane surrounding the cell containing lipopolysaccharides [43]. Phytoplasmas are strictly neither Gram-positive nor Gram-negative in terms of their membrane composition [15], but they are closely related to the non-sterol-requiring acholeplasmas [44]. To choose the best option for phytoplasmas, SignalP programs were tested both in Gram-positive and Gram-negative modes on the positive dataset; better retrieval of phytoplasma effectors was achieved in the Gram-positive mode. This is consistent with phytoplasmas’ phylogeny, since they belong to the Mollicutes class—cell wall-less microorganisms derived from a Bacillus/Clostridium-like ancestor [45,46]. In other words, phytoplasmas are derived from Gram-positive bacteria but they lack the cell wall.

The mechanisms for delivering effectors are also different between Gram-negative and Gram-positive bacteria. Gram-negative bacteria have type III, type IV and type VI secretion systems that form hollow tubes through which effectors are directly translocated from the bacterial cytosol directly into the cytosol of host cells [47]. In the case of Gram-positive bacteria, they predominantly use the Sec-dependent pathway for effector delivery [48]. Therefore, the SignalP program is suitable for the identification of phytoplasma effectors, and different versions of this program have been used [11,13,15]. Here, a comparison of pipelines only differing in the version 4.1 or 5.0 of SignalP showed that the specificity (proportion of negatives that were correctly identified) of SignalP v5.0 was higher than that of SignalP v4.1, but sensitivity of SignalP v5.0 (proportion of positives that are correctly identified) was lower; simply put, v5.0 excluded more true effectors. Similar results were found by Tan et al. (2021) [14] with SignalP v5.0 identifying less false positives in ‘*Ca*. Phytoplasma aurantifolia’ than SignalP v4.1, but SignalP v5.0 also identified a smaller number of effectors. In order to distinguish the performance of the pipelines evaluated here, the F1 scores (these measure the success of the pipelines; best value at 1 and the worst score at 0) were calculated. F1 scores were higher for pipelines that harbor SignalP v4.1 (Table 3 and Table 4), supporting this version for the search for effectors in the phytoplasma proteomes. However, none of the pipelines were able to retrieve all sequences from the positive dataset. The best pipeline (pipeline 1, indicated in Table 3 with a F1 score of 0.91) excluded three true effectors. We reasoned that the best pipeline should be able to recover all or most of the effectors, even if a large number of false positives are initially recovered, followed by the elimination of the false positives present.

During the in silico characterization of the effectors, we realized that some of them were identified by homology because selection based on common characteristics (having signal peptide and not having TMD) would exclude them. For example, protein ID A0A859I9H9 (GenBank QKX95313.1) from the rapeseed phyllody phytoplasma is homologous to the effector SAP01 from the phytoplasma AY-WB, but the mature protein (after in silico removal of the signal peptide) has a TMD according to TMHMM. Therefore, to improve the pipeline’s sensitivity, a Blastp step was included using the phytoplasma effectors’ positive dataset as query. Subsequently, the functional annotation of known effectors and essential proteins was used to distinguish effector candidates from false positives. The hits with descriptors that match with annotations of essential proteins were ruled out.

The search for effectors based on homology was also used in the prediction of fungal and oomycete effectors by WideEffHunter and demonstrated to improve its accuracy on real tests beyond positive control [49].

This strategy showed no significant differences in the identification of effectors on the positive dataset (Table 3 and Table 4), but the combination of the different programs used in the different reports, including SignalP v4.1, TMHMM v2.0, Phobius and SecretomeP, along with the search for homologous sequences of known effectors, significantly expanded the number of effector candidates retrieved in the testing dataset (see in Table 5, pipeline 2 vs. pipeline 1; pipeline 4 vs. pipeline 3; pipeline 6 vs. pipeline 5; and pipeline 8 vs. pipeline 7). Later, the last step of the pipeline specifically eliminated FP, resulting in higher F1 scores in all cases of pipelines that include the steps of Blastp and elimination of FP. The pipeline # 2 was chosen as the best predictor and it was named “PhyEffector”. To challenge the pipeline, “PhyEffector” was applied on a number of deduced proteomes of phytoplasmas. PhyEffector identified double to triple the number of effector candidates identified by the authors of previous phytoplasma-related reports. The analyses showed that PhyEffector identified zero FP and a low number (<10) of FN, while both parameters were greater numbers in all previous reports. The F1 score for PhyEffector ranked from 0.8957 to 1, with an average value of 0.9783, evidencing that this is a robust predictor of phytoplasma effectors. The results from PhyEffector indicate that phytoplasma effectoromes have been underestimated. Larger phytoplasma effectoromes were predicted than previously reported, and these large sizes are congruent with the fact that many phytoplasma proteins are annotated as “hypothetical protein”; for example, 257 hypothetical proteins in ‘*Ca*. Phytoplasma solani’ SA-1, and 337 in ‘*Ca*. Phytoplasma australiense’ PAa [18], and 81 in Peanut Witches’-Broom Phytoplasma [13]. Hypothetical proteins are not part of the core proteins because core proteins are conserved and have known essential functions. Therefore, some or all of these hypothetical proteins may be part of phytoplasma effectoromes [15,21]. PhyEffector demonstrated the ability to identify these novel effectors at the genome level, retrieving not only classical effector candidates but also the non-classical ones.

It is important to emphasize that PhyEffector does not rank effectors based on probability. Researchers may prioritize effectors according to their own interests. Conventional predictions pay attention to homologs of known effector families [14], or to genomic location (in PMUs) [13,50] or high expression during host or insect vector infection [16,51]. Other researchers look for novel effectors, choosing candidates with non-canonical characteristics [21,52]. Regardless of the type of phytoplasma effectors that the researcher is interested in, the PhyEffector algorithm may be useful to them. PhyEffector may be found at https://github.com/Gisel-Carreon/PhyEffector. Researchers are encouraged to experimentally validate the chosen effector candidates after in silico prediction, especially the novel non-classical ones.

## 5. Conclusions

The number of validated phytoplasma effectors is still very small. A carefully constructed positive dataset was made possible through the inclusion of putative effector candidates from the UNIPROT database, avoiding high-risk false positives; over-representation/overfitting of any class of effectors was prevented by including only a few members per family (2–4, except for SAP11, which is a large family with highly divergent members). Proteins in the positive database or testing database usually shared ~50% identity with each other in the same database and between both of these databases.

The pipeline containing the SignalP v4.1 in Gram-positive mode, in combination with Phobius and TMHMM2.0, resulted in the best option to identify phytoplasma effectors. Blastp with effector protein sequences as queries improved the identification of phytoplasma effectors. All retrieved proteins were pooled, and redundant proteins were eliminated. Many false positives were effectively excluded by eliminating proteins that share annotations of conserved core proteins, while effector candidates were supported for proteins that share common annotations of phytoplasma effectors. This entire pipeline was named PhyEffector.

PhyEffector (https://github.com/Gisel-Carreon/PhyEffector) is a pipeline suitable for the identification of effectors in phytoplasma genomes, with an average F1 score of 0.9761. It is able to not only retrieve classical but also non-classical phytoplasma effectors.

## Figures and Tables

**Table 2:** Analyses of phytoplasma effectors features.

Characteristics	Number of Effectors	% of the Total *
SP **	58	90.6
Nc-SecP	3 ***	4.7
Non-secreted	3 ****	4.7
0 TMD *****	59	92.2
1 TMD	5	7.8

* Considering 64 phytoplasma effector sequences in the positive dataset; ** by SignalP v4.1; *** Considering the 6 sequences which were not recognized by SignalP v4.1 but recognized by SecretomeP v2.0; **** Results which were not recognized by SignalPv4.1 neither by SecretomeP v2.0; ***** by TMHMM v2.0 on mature proteins (without signal peptide).

**Table 3:** Comparison of Signal P v4.1 and Signal P v5.0, in Gram-positive mode, in different pipelines for the prediction of Phytoplasma effectors.

Pipeline 1 (Signalp4.1 + phobius + secretomeP2.0 + TMHMM2.0)								
Control set	Proteins	Prediction	Sen	Spe	PPV	ACC	FPR	F1 score
Positive dataset	64	61	0.93	0.89	0.89	0.91	0.10	0.91
Negative dataset	64	7						
Pipeline 2 (Signalp4.1 + phobius + secretomeP2.0 + TMHMM2.0 + BLASTP+ elimination of false positive)								
Control set	Proteins	Prediction	Sen	Spe	PPV	ACC	FPR	F1 score
Positive dataset	64	64	1	1	1	1	0	1
Negative dataset	64	0						
Pipeline 3 (Signalp4.1 + phobius + TMHMM2.0)								
Control set	Proteins	Prediction	Sen	Spe	PPV	ACC	FPR	F1 score
Positive dataset	64	59	0.92	0.92	0.92	0.92	0.07	0.92
Negative dataset	64	5						
Pipeline 4 (Signalp4.1 + phobius + TMHMM2.0 + BLASTP+ elimination of false positive)								
Control set	Proteins	Prediction	Sen	Spe	PPV	ACC	FPR	F1 score
Positive dataset	64	64	1	1	1	1	0	1
Negative dataset	64	0						
Pipeline 5 (Signalp5.0 + phobius + secretomeP2.0 + TMHMM2.0)								
Control set	Proteins	Prediction	Sen	Spe	PPV	ACC	FPR	F1 score
Positive dataset	64	55	0.85	0.95	0.94	0.90	0.04	0.90
Negative dataset	64	3						
Pipeline 6 (Signalp5.0 + phobius + secretomeP2.0 + TMHMM2.0 + BLASTP+ elimination of false positive)								
Control set	Proteins	Prediction	Sen	Spe	PPV	ACC	FPR	F1 score
Positive dataset	64	64	1	1	1	1	0	1
Negative dataset	64	0						
Pipeline 7 (Signalp5.0 + phobius + TMHMM2.0)								
Control set	Proteins	Prediction	Sen	Spe	PPV	ACC	FPR	F1 score
Positive dataset	64	52	0.81	0.98	0.98	0.89	0.01	0.88
Negative dataset	64	1						
Pipeline 8 (Signalp5.0 + phobius + TMHMM2.0 + BLASTP+ elimination of false positive)								
Control set	Proteins	Prediction	Sen	Spe	PPV	ACC	FPR	F1 score
Positive dataset	64	64	1	1	1	1	0	1
Negative dataset	64	0						

BlastP = [BlastP with the positive dataset constructed in this work (results added to positive predictions); Blast of false positives or suspected false positives against a database with annotations of known effectors (hits are included); Blast of false positives or suspected false positives against a database with annotations of metabolic essential activities (hits are discarded)]. Sen: Sensitivity; Spe: Specificity; PPV: Positive Predictive Value; ACC: Accuracy; FPR: False positive rate; F1 score: Measure of the success of binary classifier (score reaches its best value at 1, and worst score at 0).

**Table 4:** Comparison of Signal P v4.1 and Signal P v5.0, in Gram-negative mode, in different pipelines for the prediction of Phytoplasma effectors.

Pipeline 1 (Signalp4.1 + phobius + secretomeP2.0 + TMHMM2.0)								
Control set	Proteins	Prediction	Sen	Spe	PPV	ACC	FPR	F1 score
Positive dataset	64	60	0.93	0.93	0.93	0.93	0.06	0.93
Negative dataset	64	4						
Pipeline 2 (Signalp4.1 + phobius + secretomeP2.0 + TMHMM2.0 + BLASTP+ elimination of false positive)								
Control set	Proteins	Prediction	Sen	Spe	PPV	ACC	FPR	F1 score
Positive dataset	64	64	1	1	1	1	0	1
Negative dataset	64	0						
Pipeline 3 (Signalp4.1 + phobius + TMHMM2.0)								
Control set	Proteins	Prediction	Sen	Spe	PPV	ACC	FPR	F1 score
Positive dataset	64	56	0.87	0.96	0.96	0.92	0.03	0.0.91
Negative dataset	64	2						
Pipeline 4 (Signalp4.1 + phobius + TMHMM2.0 + BLASTP+ elimination of false positive)								
Control set	Proteins	Prediction	Sen	Spe	PPV	ACC	FPR	F1 score
Positive dataset	64	64	1	1	1	1	0	1
Negative dataset	64	0						
Pipeline 5 (Signalp5.0 + phobius + secretomeP2.0 + TMHMM2.0)								
Control set	Proteins	Prediction	Sen	Spe	PPV	ACC	FPR	F1 score
Positive dataset	64	49	0.76	0.95	0.94	0.85	0.04	0.84
Negative dataset	64	3						
Pipeline 6 (Signalp5.0 + phobius + secretomeP2.0 + TMHMM2.0 + BLASTP+ elimination of false positive)								
Control set	Proteins	Prediction	Sen/Rec	Spe	PPV/Prec	ACC	FPR	F1 score
Positive dataset	64	64	1	1	1	1	0	1
Negative dataset	64	0						
Pipeline 7 (Signalp5.0 + phobius + TMHMM2.0)								
Control set	Proteins	Prediction	Sen	Spe	PPV	ACC	FPR	F1 score
Positive dataset	64	45	0.70	0.98	0.97	0.84	0.01	0.81
Negative dataset	64	1						
Pipeline 8 (Signalp5.0 + phobius + TMHMM2.0 + BLASTP+ elimination of false positive)								
Control set	Proteins	Prediction	Sen	Spe	PPV	ACC	FPR	F1 score
Positive dataset	64	64	1	1	1	1	0	1
Negative dataset	64	0						

BlastP = [BlastP with the positive dataset constructed in this work (results added to positive predictions); Blast of false positives or suspected false positives against a database with annotations of known effectors (hits are included); Blast of false positives or suspected false positives against a database with annotations of metabolic essential activities (hits are discarded)]. Sen: Sensitivity; Spe: Specificity; PPV: Positive Predictive Value; ACC: Accuracy; FPR: False positive rate; F1 score: Measure of the success of binary classifier (score reaches its best value at 1, and worst score at 0).

**Table 5:** Prediction of Phytoplasma effectors using different pipelines on a testing dataset.

Pipeline 1 (Signalp4.1 + phobius + secretomeP2.0 + TMHMM2.0)								
Set	Num. Proteins	Prediction	Sen	Spe	PPV	ACC	FPR	F1 score
Testing set	226	181	0.80	0.84	0.83	0.82	0.15	0.81
Negative set	226	35						
Pipeline 2 (Signalp4.1 + phobius + secretomeP2.0 + TMHMM2.0 + BLASTP+ elimination of false positive)								
Set	Num. Proteins	Prediction	Sen	Spe	PPV	ACC	FPR	F1 score
Testing set	226	189	0.83	0.99	0.99	0.91	0.004	0.90
Negative set	226	1						
Pipeline 3 (Signalp4.1 + phobius + TMHMM2.0)								
Set	Num. Proteins	Prediction	Sen	Spe	PPV	ACC	FPR	F1 score
Testing set	226	158	0.69	0.94	0.92	0.82	0.05	0.79
Negative set	226	13						
Pipeline 4 (Signalp4.1 + phobius + TMHMM2.0 + BLASTP+ elimination of false positive)								
Set	Num. Proteins	Prediction	Sen	Spe	PPV	ACC	FPR	F1 score
Testing set	226	172	0.76	0.99	0.98	0.87	0.008	0.86
Negative set	226	1						
Pipeline 5 (Signalp5.0 + phobius + secretomeP2.0 + TMHMM2.0)								
Set	Num. Proteins	Prediction	Sen	Spe	PPV	ACC	FPR	F1 score
Testing set	226	127	0.56	0.87	0.81	0.71	0.12	0.66
Negative set	226	29						
Pipeline 6 (Signalp5.0 + phobius + secretomeP2.0 + TMHMM2.0 + BLASTP+ elimination of false positive)								
Set	Num. Proteins	Prediction	Sen	Spe	PPV	ACC	FPR	F1 score
Testing set	226	138	0.61	0.98	0.97	0.79	0.01	0.75
Negative set	226	1						
Pipeline 7 (Signalp5.0 + phobius + TMHMM2.0)								
Set	Num. Proteins	Prediction	Sen	Spe	PPV	ACC	FPR	F1 score
Testing set	226	89	0.39	0.93	0.86	0.66	0.06	0.54
Negative set	226	12						
Pipeline 8 (Signalp5.0 + phobius + TMHMM2.0 + BLASTP+ elimination of false positive)								
Set	Num. Proteins	Prediction	Sen	Spe	PPV	ACC	FPR	F1 score
Testing set	226	106	0.46	0.99	0.98	0.73	0.008	0.63
Negative set	226	2						

Pipelines 1–4, SignalP is in Gram-positive mode; Pipelines 5–8, SignalP is in Gram-negative mode. BlastP = [BlastP with the positive dataset constructed in this work (results added to positive predictions); Blast of false positives or suspected false positives against a database with annotations of known effectors (hits are included); Blast of false positives or suspected false positives against a database with annotations of metabolic essential activities (hits are discarded)]. Sen: Sensitivity; Spe: Specificity; PPV: Positive Predictive Value; ACC: Accuracy; FPR: False positive rate; F1 score: Measure of the success of binary classifier (score reaches its best value at 1, and worst score at 0).

**Table 6:** Effector prediction by PhyEffector on phytoplasma genomes.

Phytoplasma	Effectors Predicted by the Authors	Pipeline Used for Effector Prediction *	Reference	PhyEffector Prediction	Shared Candidates	Unshared Candidates	False Negatives	False Positives	F1 Score ***
‘*Ca*. Phytoplasma mali’	31	SignalP v4.0, for SP, and then TMHMM v2.0 on mature protein sequence without the SP	[13]	49	18	A = 13 P = 31	A = 31 P = 6	A = 7 P = 0	0.9423
‘*Ca*. Phytoplasma australiense’	61	SignalP v4.0, for SP, and then TMHMM v2.0 on mature protein sequence without the SP	[13]	89	43	A = 18 P = 46	A = 46 P = 10	A = 8 P = 0	0.9518
‘*Ca*. Phytoplasma asteris’ (AY-WB)	58	SignalP v4.0, for SP, and then TMHMM v2.0 on mature protein sequence without the SP	[13]	73	35	A = 23 P = 38	A = 38 P = 17	A = 6 P = 0	0.8957
‘*Ca*. Phytoplasma asteris’ OY-M	65	SignalP v4.0, for SP, and then TMHMM v2.0 on mature protein sequence without the SP	[13]	85	54	A = 11 P = 50	A = 50 P = 7	A = 4 P = 0	0.9674
‘*Ca*. Phytoplasma solani’ strain SA-1	38	SignalP v3.0, for SP, and then TMHMM v2.0 on mature protein sequence without the SP	[18]	96	26	A = 12 P = 83	A = 83 P = 4	A = 8 P = 0	0.9819
‘*Ca*. Phytoplasma asteris’ AYWB	33	SignalP v3.0, for SP, and then TMHMM v2.0 on mature protein sequence without the SP	[11]	73	23	A = 10 P = 40	A = 40 P = 5	A = 5 P = 0	0.9668
‘*Ca*. Phytoplasma. asteris’ NJAY	23	SignalP v5.0, for SP, and then TMHMM v2.0 on mature protein sequence without the SP	[11]	95	18	A = 5 P = 77	A = 77 P = 0	A = 5 P = 0	1
‘*Ca*. Phytoplasma asteris’ WEID	17	SignalP v5.0, for SP, and then TMHMM v2.0 on mature protein sequence without the SP	[11]	64	11	A = 6 P = 53	A = 53 P = 1	A = 5 P = 0	0.9922
‘*Ca*. Phytoplasma asteris’ OY-M	37	SignalP v5.0, for SP, and then TMHMM v2.0 on mature protein sequence without the SP	[11]	84	15	A = 22 P = 69	A = 84 P = 5	A = 17 P = 0	0.9710
‘*Ca*. Phytoplasma asteris’ OY-V	36	SignalP v5.0, for SP, and then TMHMM v2.0 on mature protein sequence without the SP	[11]	87	24	A = 12 P = 63	A = 129 P = 4	A = 8 P = 0	0.9870
‘*Ca*. Phytoplasma asteris’ DY2014	45	SignalP v5.0, for SP, and then TMHMM v2.0 on mature protein sequence without the SP	[11]	97	31	A = 14 P = 52	A = 52 P = 2	A = 12 P = 0	0.9944
‘*Ca*. Phytoplasma asteris’ MBP-M3	13	SignalP v5.0, for SP, and then TMHMM v2.0 on mature protein sequence without the SP	[11]	64	9	A = 4 P = 51	A = 56 P = 0	A = 4 P = 0	1
‘*Ca*. Phytoplasma asteris’ De Villa	10	SignalP v5.0, for SP, and then TMHMM v2.0 on mature protein sequence without the SP	[11]	55	5	A = 5 P = 50	A = 50 P = 1	A = 4 P = 0	0.9909
‘*Ca*. Phytoplasma asteris’ LD1	14	SignalP v5.0, for SP, and then TMHMM v2.0 on mature protein sequence without the SP	[11]	60	9	A = 5 P = 46	A = 56 P = 1	A = 4 P = 0	0.9923
‘*Ca*. Phytoplasma asteris’ CYP	21	SignalP v5.0, for SP, and then TMHMM v2.0 on mature protein sequence without the SP	[11]	91	14	A = 7 P = 77	A = 77 P = 0	A = 7 P = 0	1
‘*Ca*. Phytoplasma asteris’ TW1	19	SignalP v5.0, for SP, and then TMHMM v2.0 on mature protein sequence without the SP	[11]	57	14	A = 5 P = 38	A = 51 P = 2	A = 3 P = 0	0.9827
‘*Ca*. Phytoplasma hytoplasma aurantifolia’	98 SignalP v4.1	Comparison of SignalP v4.1 and SignalP v5.0; the former retrieved ~ 70 false positives.	[14]	93	53	A = 45 P = 40	A = 40 P = 8	A = 37 P = 0	0.9587
‘*Ca*. Phytoplasma aurantifolia’	27 ** SignalP v5.0	Comparison of SignalP v4.1 and SignalP v5.0; the former retrieved ~ 70 false positives.	[14]	93	20	A = 7 P = 73	A = 73 P = 5	A = 2 P = 0	0.9738
‘*Ca*. Phytoplasma vitis’ (Flavescence dorée)	17	SignalP v5.0 and Phobius. Effectors with transmembrane domains (TMDs) were also identified.	[12]	41	6	A = 11 P = 35	A = 35 P = 2	A = 9 P = 0	0.9791
‘*Ca*. Phytoplasma ziziphi’ (Jujube witches’-broom Phytoplasma)	8 (Zaofeng1 to Zaofeng8).	Signal peptide by SignalP 4.1 and TMDs by the TMHMM 2.0. Potential mobile units (PMUs) were identified by the presence of flanking tra5 insertion sequences and DNA replication genes (dnaG, dnaB, ssb, tmk). Secreted proteins harbored in PMUs were identified as JWB phytoplasma putative effectors.	[35]	87	5	A = 3 P = 82	A = 89 P = 1	A = 2 P = 0	0.9942

* SignalP v3.0, v4.0, v5.0 were used on the Gram-positive bacteria mode. A = authors; P = PhyEffector. ** Authors recognized this number as the correct prediction. *** F1 score calculated using the effector prediction by PhyEffector in each genome.

## Data Availability

All data are provided in the manuscript and Supplementary Materials. Table S1: Positive data set; Table S2: Negative data set; Table S3: List of functional annotation of Phytoplasma effectors; Table S4: Testing set and Table S5: Second negative set. PhyEffector algorithm is available at https://github.com/Gisel-Carreon/PhyEffector.

## Supplementary Materials

The following supporting information can be downloaded at: https://www.mdpi.com/article/10.3390/biomimetics8070550/s1. Table S1. Positive dataset. The list comprises 64 phytoplasma protein sequences (11 true validated effectors and 53 selected effector accessions from UNIPROT); Table S2. Negative dataset. The list comprises 64 diverse phytoplasma core protein (essential metabolic proteins); Table S3. List of functional annotation of Phytoplasma effectors; Table S4. Testing dataset. The list comprises 226 phytoplasma protein sequences (10 true validated effectors and 216 selected effector accessions from UNIPROT, different and divergent than proteins comprising the positive dataset); Table S5. Negative dataset. This second negative dataset comprises 226 phytoplasma core protein, different from proteins in the other negative dataset.
