# Supplementary material for: PhyEffector, the First Algorithm That Identifies Classical and Non-Classical Effectors in Phytoplasmas
Source: Biomimetics (Basel). 2023 Nov 17;8(7):550. doi: 10.3390/biomimetics8070550 (PMC10669590; doi:10.3390/biomimetics8070550)
Supplement: Supplementary file 1 [file biomimetics-08-00550-s001.zip › Table S3.pdf]

Table S3. List of functional annotation of Phytoplasma effectors

Tengu-su inducer
SAP1-like protein
SAP02-like protein
SAP05-like protein
SAP06-like protein
SAP08-like protein
SAP09-like protein
SAP11 effector protein
SAP19-like protein
SAP20-like protein
SAP21-like protein
SAP30-like protein
SAP34-like protein
SAP36-like protein
SAP37-like protein
SAP39-like protein
SAP40-like protein
SAP42-like protein
SAP43-like protein
SAP44-like protein
SAP45-like protein
SAP48-like protein
SAP49-like protein
SAP50-like protein
SAP53-like protein
SAP54-like protein
SAP55-like protein
SAP56-like protein
SAP59-like protein
SAP61-like protein
SAP63-like protein
SAP64-like protein
SAP65-like protein
SAP66-like protein
SAP67-like protein
SAP68-like protein
phytoplasma effector causing phyllody symptoms
Secreted effector protein containing SVM protein
Effector causing phyllody symptoms 1 (Phyl-1)
SVM family protein
Effector protein/putative effector
Putative phage integrase
MPEP-jgl1_1
PME2-sporadic
PME2ST
antigenic membrane protein (Amp)

Immunodominant membrane protein A (idpA)<br>Immunodominant membrane protein (Imp)<br>Variable membrane protein A (VmpA)<br>Secreted AYWB protein (SAP)<br>type III secretion system effector protein Candidatus<br>Conserved hypothetical protein*<br>Uncharacterized protein*<br>Hypothetical proteins*<br>No hits**

Most descriptions were found both in UNIPROT and GenBank.
Labeled with (*) were found as common description at GenBank in Blastp analysis using an amino acid sequence of a phytoplasma effector as query.
** No hits, which corresponds to specific proteins in the analysis of phytoplasma genomes in the literature.
